# Molecular Evolution of a Glioblastoma Controlled With Tumor Treating Fields and Concomitant Temozolomide

**DOI:** 10.3389/fonc.2018.00451

**Published:** 2018-10-15

**Authors:** H. Ian Robins, HuyTram N. Nguyen, Aaron Field, Steven Howard, Shahriar Salamat, Dustin A. Deming

**Affiliations:** ^1^Department of Medicine, University of Wisconsin School of Medicine and Public Health, Madison, WI, United States; ^2^Department of Neurology, University of Wisconsin School of Medicine and Public Health, Madison, WI, United States; ^3^Department of Human Oncology, University of Wisconsin School of Medicine and Public Health, Madison, WI, United States; ^4^University of Wisconsin School of Medicine and Public Health, Madison, WI, United States; ^5^Department of Radiology, University of Wisconsin School of Medicine and Public Health, Madison, WI, United States; ^6^Department of Pathology, University of Wisconsin School of Medicine and Public Health, Madison, WI, United States; ^7^Department of Oncology, McArdle Laboratory for Cancer Research, University of Wisconsin School of Medicine and Public Health, Madison, WI, United States

**Keywords:** glioblastoma, tumor treating fields, optune®, genomics, temozolomide

## Abstract

Tumor Treating Field (TTFields) therapy has demonstrated efficacy in a Phase 3 study of newly diagnosed glioblastoma (GB) following radiation (RT) and temozolomide (TMZ). We report the appearance of an isolated satellite anterior temporal lobe lesion, 2 months post primary RT/TMZ directed at the primary GB (MGMT methylated) parietal lobe lesion and one adjuvant cycle of TMZ and TTFields. The mean RT dose delivered to the temporal lobe lesion was negligible, i.e., 4.53 ± 0.95 Gy. Mapping of the generated TTFields demonstrated that both lesions were encompassed by a field intensity in a therapeutic range. The temporal lobe lesion remained under the control of TTFields up to 12 months, at which point progression on a T1 contrast MRI resulted in surgery and a definitive diagnosis of GB without MGMT methylation. The primary parietal lobe at this time was in remission. Molecular sequencing on the GB tissue from multiple time points demonstrates clonal evolution of the cancer over time and in response to treatment.

## Introduction

Tumor Treating Field (TTFields) therapy has demonstrated efficacy in a Phase 3 study of newly diagnosed glioblastoma (GB) following radiation (RT) and temozolomide (TMZ) (1), as well as in the recurrent setting (2). Interestingly, there have been no reports of TTFields therapy in GB patients who have not received prior RT. In addition, the potential mechanisms by which resistance to TTFields therapy develops has been understudied.

In the report to follow, an analysis of a satellite lesion that developed after standard RT and TMZ therapy in a newly diagnosed GB patient is presented. Therapy with TTFields had been initiated 1 month prior to the appearance of the satellite lesion. The patient was followed longitudinally with MRIs every 2 months; additional analysis of the radiation dose exposure, as well as the TTFields intensity, was performed. The differential diagnosis at the time included an MRI artifact or lesion induced by TTFields, vs. progressive disease. After 12 months, the aforementioned lesion was resected. Molecular alterations from baseline, post-progression on TTFields and following a further recurrence were assayed. The results below summarize these collective findings.

## Clinical details and data analysis

In March 2016, a 51-year old male presented with left-sided numbness and weakness. A MRI demonstrated a 35 × 25 × 29 mm partially cystic or necrotic, enhancing mass with internal hemorrhage in the right parietal lobe. Subtotal resection was accomplished in March 2016 confirming a grade 4 astrocytoma with IDH1/2 wild type, MGMT methylated, and negative 1p19q co-deletion.

**Figure 1:**
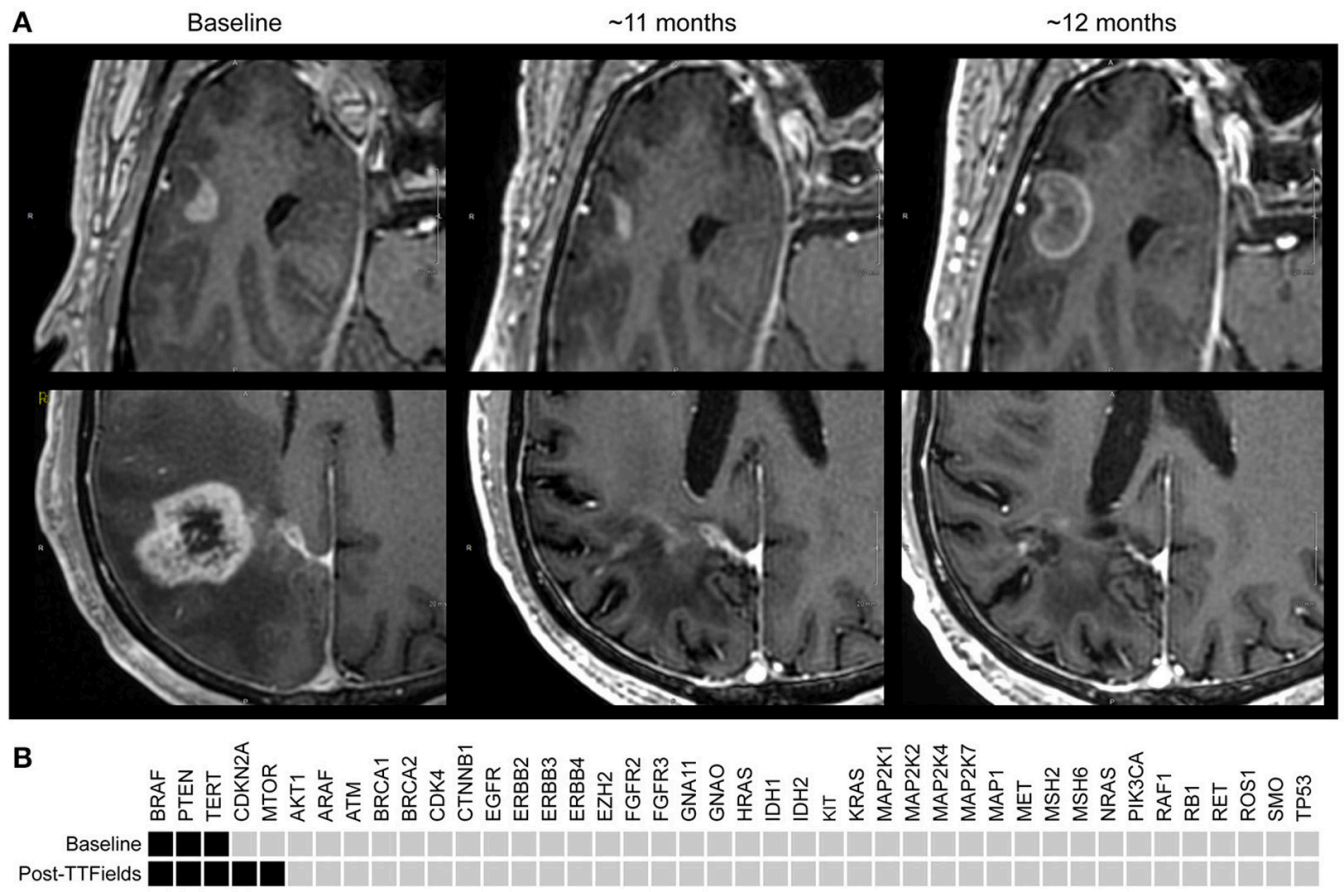
**(A)** T1+contrast MRI images: Upper panels are right temporal lobe; lower panels are corresponding right parietal images. *Baseline* (Aug. 2016) demonstrates the first appearance a temporal lobe lesion ~2 months post radiation/temozolomide; the lower panel demonstrates the primary GB. Middle section ~*11 months* later (June 2017) demonstrates slightly less enhancement of the temporal lobe lesion, and a dramatic reduction in enhancement and size of the parietal lobe lesion with decreased edema and treatment related cerebral atrophy. At ~*12 months* (Aug. 2017) the temporal lobe lesion has increased to 18 × 13 mm; the parietal lobe remains stable and in remission. **(B)** StrataNGS cancer hotspot sequencing was performed on the resection of the primary parietal lobe lesion, which possessed mutations in *BRAF* (V600E), *PTEN* (319fs), and the *TERT* promoter (C228T). Following progression on TTFields, the separate anterior temporal lesion was resected and demonstrated *BRAF, PTEN*, and *TERT* alterations, and the acquisition of a deep deletion of *CDK2NA* and an activating mutation in *mTOR* (V2006I). No other pathologic alterations were identified in the remaining 47 genes of the 88 genes assessed.

Standard radio-chemotherapy was completed in June 2016 (3), including daily TMZ with a total of 60 Gy radiation give in 30 fractions; adjuvant TMZ began in July 2016. TTFields therapy (1) was initiated in July 2016 and continued until August 2017. A post-radiation MRI was done in August 2016, showing increased thickness of the residual enhancing region in the right parietal lobe in addition to a new lesion in the right middle temporal gyrus (Figure 1).

Changes of residual tumor in the right parietal lobe was presumed to be progression vs. pseudo-progression, and the patient continued with six cycles of adjuvant TMZ, which was completed in December 2016. The changes in the parietal lobe lesion resolved over time, confirming pseudo-progression. In spite of the appearance of the temporal lobe lesion, it was decided to continue therapy with both TMZ and TTFields (with frequent monitoring), as the possibility of an artifact of TTFields therapy and/or an unusual form pseudo-progression was raised.

**Figure 2:**
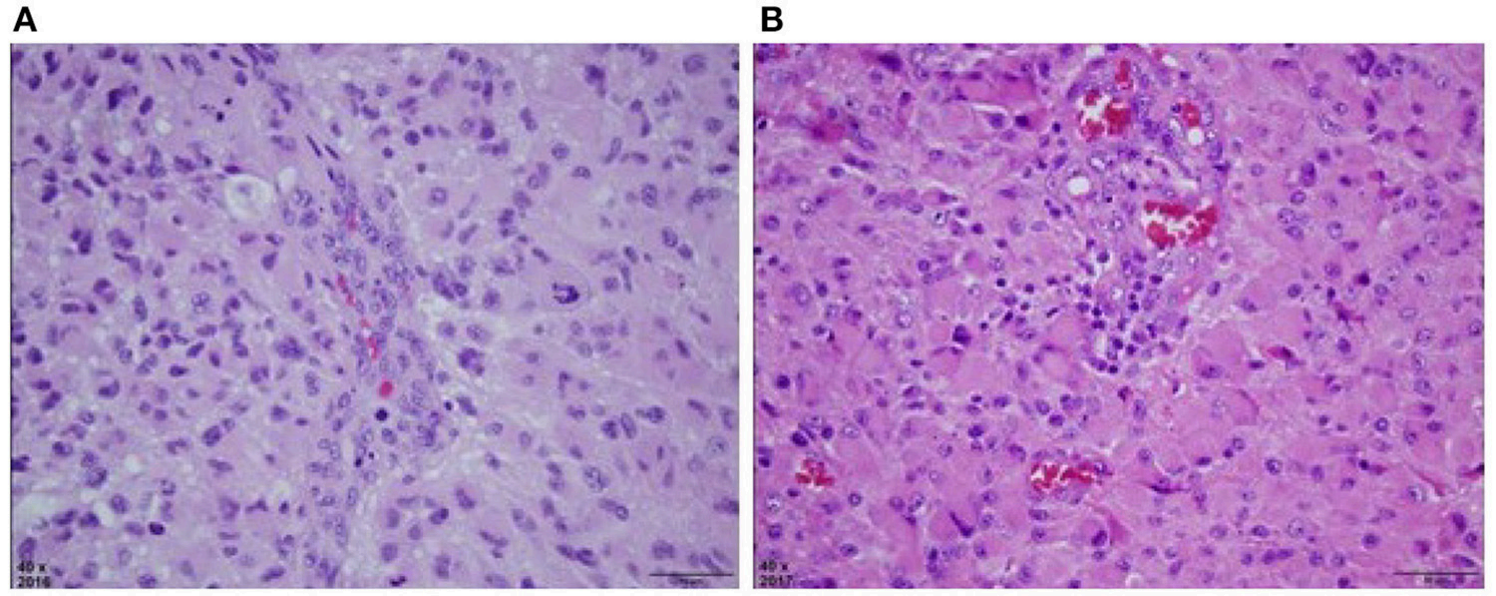
**(A)** H&E stained section of right parietal tumor at original magnification of 40x, reveals a densely cellular astrocytic neoplasm with nuclear atypia, mitosis, and vascular endothelial proliferation. Palisaded necrosis was also present but not shown in this field. **(B)** H&E stained section of right temporal mass at original magnification of 40x, also reveals a densely cellular astrocytic neoplasm with slightly more gemistocytic features, nuclear atypia, mitosis, and vascular endothelial proliferation that was similar to the previously resected tumor. This material lacked necrosis.

**Figure 3:**
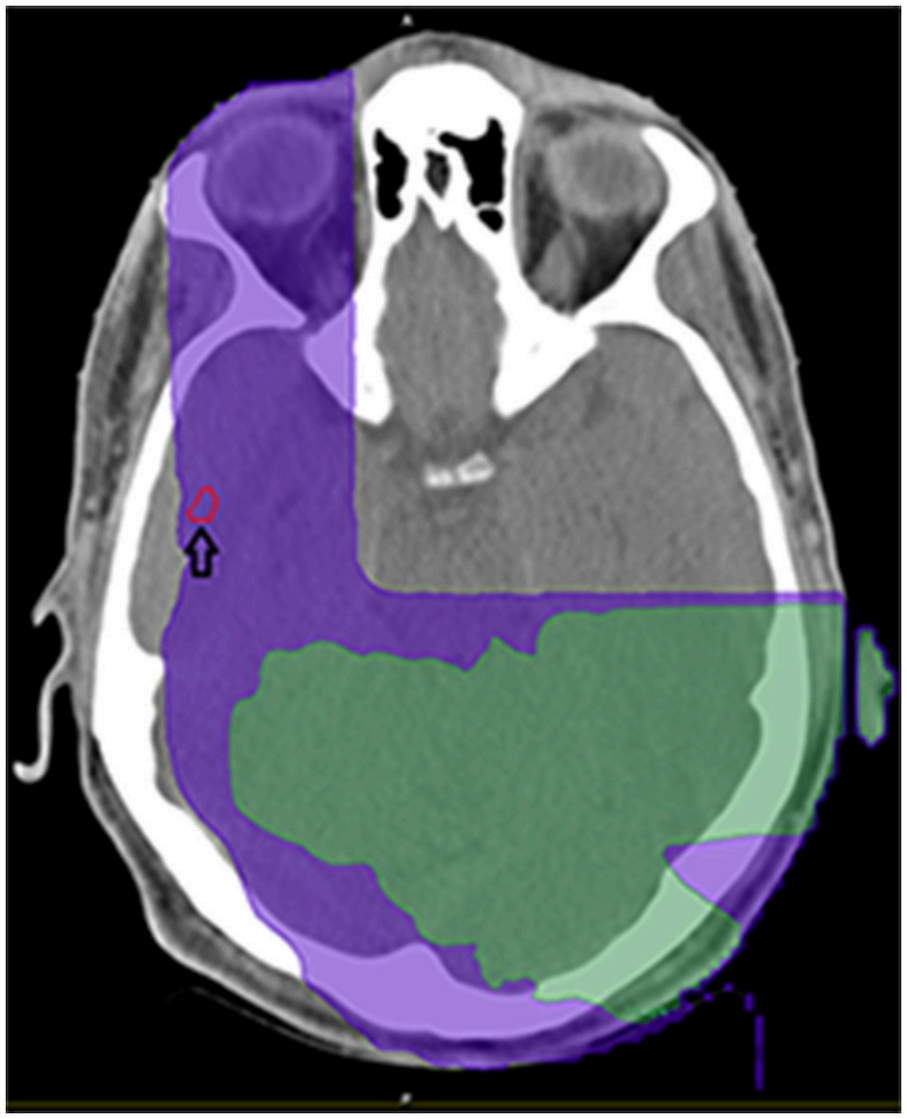
Demonstration of isodose cloud for temporal lobe lesion (see arrow). Purple denotes 5Gy isodose; green denotes 8.57 Gy isodose.

On a series of follow-up MRIs from August 2016 to August 2017, the initial parietal lobe lesion regressed with adjuvant TMZ and appeared stable on both T1+contrast and T2/FLAIR MRIs. The new enhancing lesion in the temporal lobe (during adjuvant TTFields/TMZ therapy) decreased from 9 to 7.7 mm in diameter with decreasing enhancement from August 2016 to November 2016 (Figure 1), and stayed stable on bi-monthly follow up MRIs until August 2017. At this time the temporal lobe lesion was at 17.9 mm in diameter (on T1+contrast); the parietal lobe lesion was essentially resolved, confirming pseudo-progression of this tumor (Figure 1). T2/FLAIR images showed abnormality with an area of restricted diffusion and peripheral rim enhancement in the region of the right temporal lobe lesion. A gross total resection of the temporal lesion was achieved in August 2017, confirming a grade 4 astrocytoma, with wild type IDH1/2, unmethylated MGMT, and negative 1p19q co-deletion (Figure 2). The mean prior radiation dose for this temporal lesion was determined to be 4.53 Gy ± 0.95 Gy (5.7 Gy max; 3.5 Gy min; volume 0.1 mL). An isodose cloud is depicted (Figure 3). The lesion was 2.5 cm away from the edge of the planning target volume treated to full dose (46 Gy; center lesion dose 60 Gy).

The patient was then treated (September 2017–November 2017) with radiation (60 Gy in 30 fractions), targeting the temporal lobe resection cavity. An MRI in January 2018 demonstrated a possible new nodule (0.7 × 0.7 cm) on the edge of the resection cavity. A subsequent MRI in February 2018 confirmed progression with an increase in the aforementioned nodule to 1.4 × 1.8 cm. In March 2018, the patient underwent reoperation with a gross total resection as part of the TOCA 5 Tocagen Inc. clinical trial and was randomized to the control arm post-operatively. He started bevacizumab therapy in April 2018 which maintained his surgically obtained complete remission until relapse in August 2018.

Molecular analyses demonstrate that at resection of the primary parietal lobe lesion this cancer possessed mutations in *BRAF* (V600E), *PTEN* (319fs), and the *TERT* promoter (C228T). Following progression on TTFields, the separate anterior temporal lesion was resected. This lesion possessed these identical *BRAF, PTEN*, and *TERT* alterations, and was also found to possess a deep deletion of *CDK2NA* and an activating mutation in *mTOR* (V2006I). The lesion in the anterior temporal lobe that recurred following radiation was also sequenced following resection. This lesion was hypocellular, and similar to the prior resection exhibited mitosis, nuclear atypia and no necrosis; the same *BRAF, mTOR*, and *TERT* abnormalities were still able to be observed. No new alterations were detectable in this sample.

**Figure 4:**
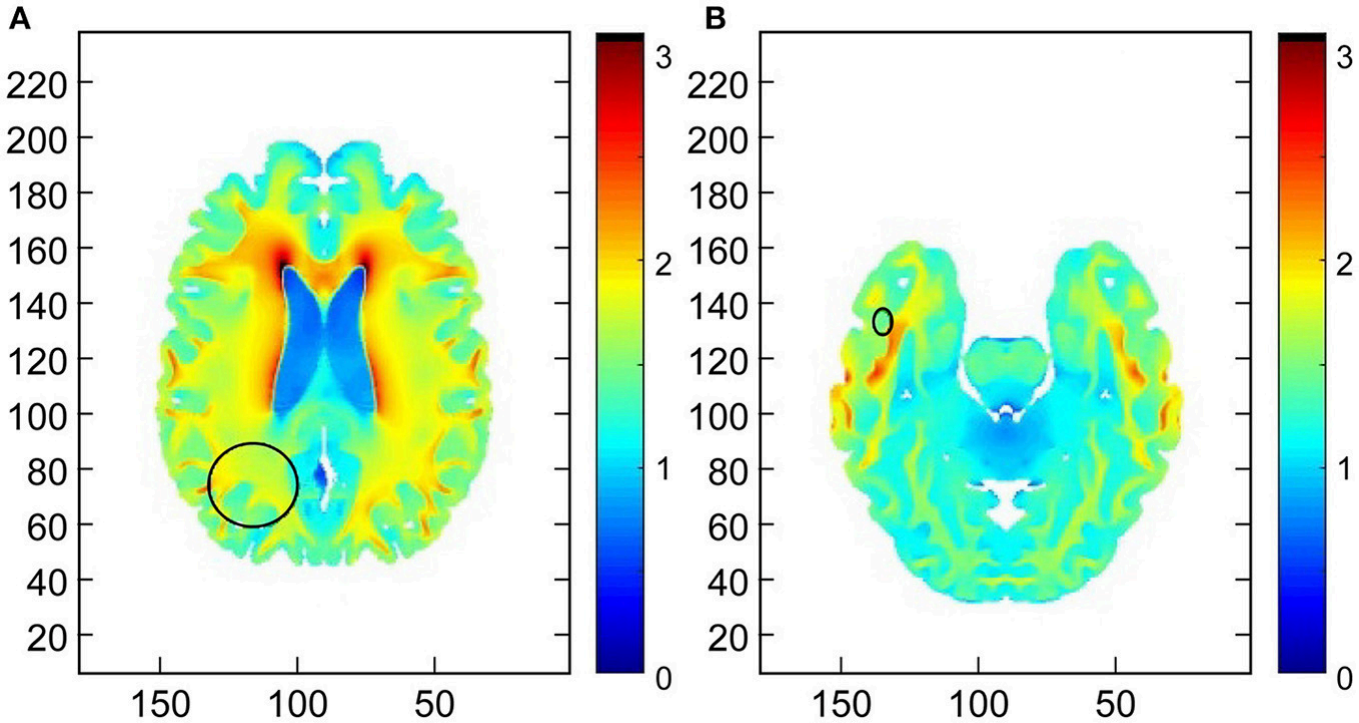
Demonstration of the field intensity distribution in axial slices through the centers of the **(A)** primary right parietal lobe lesion and **(B)** the right temporal lobe secondary lesion. The median field intensity in the region of the primary lesion was 1.7 V/cm (mean of 1.66 V/cm). In the region of the secondary lesion the median intensity was 1.48 V/cm (mean of 1.56 V/cm). This suggests that TTFields intensities around both lesions exceeded the therapeutic threshold of 1 V/cm.

Additionally, a retrospective analysis demonstrated the TTFields intensity was in a therapeutic range for both the parietal lobe and temporal lobe lesion, i.e., 1 V/cm (Figure 4).

## Methods

### Bio-marker testing

MGMT testing performed by LabCorp, NC; IDH testing done by PCR, UW Health Clinical labs, WI, 1p19q testing by FISH, Wisconsin State Laboratory of Hygiene.

### Determination of radiation dose

Using image registration software (Mim Vistag Cleveland, OH) that imports the radiation dose, the axial contrast-enhanced 3D T1-weighted images (T1 3D-SPGR Bravo, GE Healthcare, Waukesha, WI) were fused into a coordinate system of the treatment planning CT. A region of interest was drawn around the anterior temporal lobe lesion, and dosimetric analysis revealed the prior RT dose to the lesion.

### Mapping of TTFields intensity

In order to estimate field intensity distributions within the lesions, numerical simulations were performed using finite differences Time Domain (FDTD) calculations and a realistic head model as described in Wenger et al. (4). Briefly, a realistic head computational model of a healthy male was created and scaled to match the dimensions the patient's head. Transducer arrays for the delivery of TTFields were positioned on the head model to mimic the personalized transducer array layout that was placed on the patient. In order to establish whether or not TTFields were delivered at therapeutic levels to the tumors, ellipsoidal regions approximately encompassing the lesions were manually marked on the field intensity maps. The field was considered to deliver TTFields at therapeutic levels to the lesion if the median field intensity within the respective ellipsoid exceeded 1 V/cm (5).

### Strata oncology hot spot sequencing

Patient samples were sequenced through STRATA Oncology CLIA-certified laboratory using the StrataNGS platform. This panel covers 88 genes and examines predefined variants including single and multinucleotide alterations, small insertions/deletions, fusions, exon skipping mutations, copy number variation, and microsatellite instability (www.strataoncology.com).

## Discussion

In this report, we present the first instance of a grade 4 astrocytoma controlled by systemic TMZ and TTFields, with negligible radiation exposure. The patient's initial parietal lobe lesion was MGMT methylated, not IDH mutated, and not 1p19q deleted; the resected temporal lobe lesion was similar histologically, but was not MGMT methylated. Based on the MRIs between June 28th to August 14th, 2016 (Figure 1), the volume doubling time was calculated (6) as 14 days for the temporal tumor.

As the temporal lobe tumor appeared after initial concurrent radiation and TMZ treatment, this tumor may have been TMZ resistant, which is consistent with the absence of methylation on MGMT promoter. Alternatively, a resistant TMZ clone may have evolved over time. The initial radiation field was reconstructed, showing that the temporal lobe was exposed to minimal radiation at the time, 4.53 ± 0.95 Gy. This region, however, was within the TTFields effective region, suggesting that the suppression of tumor growth from August 2016 to 2017 was under the control of adjuvant TMZ and/or TTFields.

The original plan for the placement of Optune™ arrays using the NovoTAL™ methodology (7) targeted the right parietal lesion. It was not intuitively obvious that the field distribution in the temporal lobe region would be sufficiently high to have a therapeutic effect. Hence, numerical simulations (Figure 4) were performed; the simulations demonstrate that the field intensity delivered to both lesions was at therapeutic levels (>1 V/cm). Taken collectively, these data support the efficacy of TTFields in a newly diagnosed GB regarding a lesion that received a negligible dose of ionizing radiation. The contribution of adjuvant TMZ in controlling this lesion is indeterminate as discussed above.

Based on the molecular sequencing we can see that the cells within the anterior temporal lobe lesion developed from cells in the original primary parietal lobe lesions as the exact alterations were identified in both instances. The additional alterations identified presumably arose through clonal selection. While many factors could have potentially played into this selection process, we propose that it is quite plausible that the activating mutation in mTOR and/or the deep loss of CDKN2A could be inducing the resistance to TTFields therapy.

Over the last few years, new mechanistic insights have been gained into the anti-cancer effects of TTFields. These potential mechanisms of action include disruption of key cellular functions, such as mitosis, DNA repair, mitochondrial function, and the folded protein response, leading to the induction of cellular stress, autophagy and apoptosis (5, 8–10). TTFields has also been implicated in enhancing the immune response through the induction of immunogenic cell death and modulation of antigen presentation (11). Loss of CDKN2A could lead to cell cycle dysregulation and mTOR activation could lead to inhibition of autophagy, apoptosis, and enhance cell proliferation overcoming some of the potential mediators of response to TTFields (12, 13). In addition, activation of the PI3K/AKT/mTOR signaling pathway has been associated with immune suppressive properties, including the up-regulation of the PD-L1 immune checkpoint.

In summary, this report provides evidence that TTFields may offer prolonged therapeutic benefit for some patients with recurrent GB. The molecular analysis of this patient's cancer over time provides potential insight to mechanisms by which resistance to TTFields might occur. This work also raises several interesting questions about how clonal evolution and spread through the central nervous system occurs, whether targeting therapies, such as mTOR or BRAF inhibitors, could be used in settings like this, and whether more routine molecular profiling should be obtained for patients with GB. Clearly, as we learn more about the biology of individual patients with GB this will lend itself to more precision-based treatment strategies for patients.

## Ethics statement

This is a case report regarding a patient treated with FDA approved standard of care treatment. Informed consent was obtained.

## Author contributions

HN contributed to the clinical history review, literature review, and manuscript preparation. SH evaluated radiation dosing and reviewed the manuscript. SS reviewed pathology. AF reviewed the manuscript and MRI scans. HR identified the patient, reviewed the clinical history and the literature, and prepared the manuscript. DD performed the genomic analyses and edited the manuscript.

### Conflict of interest statement

HR and DD have support for laboratory research from Novocure, Ltd. The remaining authors declare that the research was conducted in the absence of any commercial or financial relationships that could be construed as a potential conflict of interest.
